# Wounding-Related Signaling Is Integrated within the Auxin-Response Framework to Induce Adventitious Rooting in Chestnut

**DOI:** 10.3390/genes15030388

**Published:** 2024-03-21

**Authors:** Ricardo Castro-Camba, Jesús Mª Vielba, Saleta Rico, Purificación Covelo, Mª José Cernadas, Nieves Vidal, Conchi Sánchez

**Affiliations:** Department of Plant Production, Misión Biológica de Galicia (CSIC), Avda de Vigo s/n, 15705 Santiago de Compostela, Spain; ricardo.castro@mbg.csic.es (R.C.-C.); jmvielba@mbg.csic.es (J.M.V.); saleta@mbg.csic.es (S.R.); pcovelo@mbg.csic.es (P.C.); cernadas@mbg.csic.es (M.J.C.); nieves@mbg.csic.es (N.V.)

**Keywords:** adventitious roots, auxin, callus, chestnut, microshoots, transcriptomics, wounding

## Abstract

Wounding and exogenous auxin are needed to induce adventitious roots in chestnut microshoots. However, the specific inductive role of wounding has not been characterized in this species. In the present work, two main goals were established: First, we prompted to optimize exogenous auxin treatments to improve the overall health status of the shoots at the end of the rooting cycle. Second, we developed a time-series transcriptomic analysis to compare gene expression in response to wounding alone and wounding plus auxin, focusing on the early events within the first days after treatments. Results suggest that the expression of many genes involved in the rooting process is under direct or indirect control of both stimuli. However, specific levels of expression of relevant genes are only attained when both treatments are applied simultaneously, leading to the successful development of roots. In this sense, we have identified four transcription factors upregulated by auxin (*CsLBD16*, *CsERF113*, *Cs22D* and *CsIAA6*), with some of them also being induced by wounding. The highest expression levels of these genes occurred when wounding and auxin treatments were applied simultaneously, correlating with the rooting response of the shoots. The results of this work clarify the genetic nature of the wounding response in chestnut, its relation to adventitious rooting, and might be helpful in the development of more specific protocols for the vegetative propagation of this species.

## 1. Introduction

The formation of adventitious roots (ARs) is essential for the vegetative propagation of forest, horticultural and crop species. By means of this asexual reproduction process, interesting or profitable genotypes can be multiplied, shortening the time needed to improve genotypes by breeding while avoiding the genetic variability inherent to sexual reproduction. However, adventitious rooting is influenced by many inner and outer factors, including nutrient availability, light conditions, genotype, temperature, ontogenetic state, etc. [1,2,3,4]. Among those factors, hormone homeostasis, and particularly auxin signaling and its crosstalk with other growth regulators, emerge as central players in the induction and development of ARs [5]. Indeed, hormones are believed to work by integrating endogenous and exogenous cues into specific gene signaling pathways that directly impact rooting processes [6].

Chestnut (*Castanea sativa* Mill.) is a relevant forest species that has significant cultural, ecological and economic relevance in several European countries. It is considered a hard-to-root species, as is the case for many other members of the Fagaceae family. As with other woody species, a strong dependency on the ability to root is linked to the genotype, while the ontogenetic state of the material is also relevant: mature tissues usually show low rooting responses [4]. Therefore, juvenile-like material collected from mature trees is preferentially used for vegetative proliferation. For the successful propagation of chestnut cuttings and microshoots, two main stimuli are needed, wounding and the exogenous application of auxin, mainly in the form of Indole-butyric acid (IBA). Wound healing and tissue regeneration are believed to be two different, although deeply connected, processes whose molecular basis is beginning to be clarified [7]. The response of chestnut cuttings to wounding stress is the formation of a callus structure, a strategy that prevents the loss of resources as well as the entrance of pathogens [8]. Nonetheless, auxin induction of roots can also proceed through the intermediate formation of a callus structure, which might decrease the quality of the generated rooting system and hinder the connection between the newly formed organ and the vascular bundles. In chestnut, different concentrations of auxin are used to induce ARs, with treatments spanning from seconds to days. It has been shown that excessive concentrations of auxin can result in rooted microshoots with callus, apical drought and poor health status [9]. Therefore, optimization of root induction protocols is required to improve the quality of the rooted plants. On the other hand, little is known about the molecular basis of the wounding response in this species, the molecular mechanisms involved in the formation of callus and how they are related to the rooting process. This knowledge might help understand the specific role of both stimuli and design more accurate protocols for the vegetative propagation of chestnut.

In the present work, several concentrations of auxin were tested in chestnut juvenile microshoots to define the optimal treatment considering both rooting rate and shoot health status. Then, a transcriptomics time-series analysis was developed to depict the genetic responses of chestnut to wounding and wounding plus auxin in order to characterize the molecular processes that are triggered by these two stimuli. Particularly, we focused on the early stages after the beginning of the treatments, where specific cells within the base of the stems might be responsive and able to trigger a cellular reprogramming driving the initiation of roots [10,11]. The results obtained suggest that wounding triggers a molecular response that paves the way for the rooting process, but several relevant genes fall short of the expression level needed to induce the formation of the roots. However, in the presence of even low exogenous auxin concentrations, those genes attain the necessary levels to govern the induction of ARs. Particularly, the combined effect of wounding and auxin significantly induced the expression of *CsLBD16*, *CsIAA6*, *Cs22D* and *CsERF113*, allowing them to influence the expression of several other genes, which seems to be a key element in the acquisition of rooting competence. Meanwhile, wounding alone induced the expression of *CsERF113* and *CsIAA6*, but at lower levels that could not trigger a rooting response. Overall, the present results suggest that auxin treatment amplifies the expression of genes that are also induced by wounding, leading to accurate gene expression levels for the induction of roots.

## 2. Materials and Methods

### 2.1. Plant Material, Wounding and Rooting Treatments

Juvenile-like microshoots obtained from basal sprouts of a field-grown chestnut (*C. sativa* Mill.) tree were used as plant material. The line was established more than three decades ago, and it was named P2BS [12]. Microshoots were grown in vitro in Gresshoff and Doy medium [13], supplemented with 0.075 mg L^−1^ of benzyladenine (BA), 30 g L^−1^ of sucrose and 7 g L^−1^ of Bacto Agar, and subcultured every four weeks. At the end of the growing period, the basal callus was eliminated using a scalpel, and the shoots were used for a new growing cycle or for wounding and rooting experiments. In the case of wounding samples (W), after elimination of the basal callus, microshoots were placed in GD medium with macronutrients reduced to 1/3 and kept in the dark for five days, when they were transferred to normal photoperiod conditions (16 h light/8 h darkness). For the wounding plus auxin samples (W + IBA), microshoots deprived of basal callus were placed in 1/3 strength GD medium supplemented with different IBA concentrations (2.5, 5, 10, 12.5 and 25 µm), kept in dark conditions for five days, and then transferred to hormone-free medium under normal photoperiod. Wounding and rooting experiments were carried out with 6 explants per glass jar, three jars per experiment, and three repeats per treatment (*n* = 54). At the end of the rooting period, the following parameters were assessed: percentage of rooted shoots, root number, mean root length of the longest root, presence of shoot tip necrosis (%), resumption of shoot growth and basal callus formation. Resumption of shoot growth represents the percentage of rooted shoots showing active growth either from the apical shoot or from lateral buds when the shoot apex dries up. Rooting kinetics were also evaluated during the rooting process.

Data normality was assessed using the Shapiro–Wilk test, and the assumption of homoskedasticity was evaluated using the Levene test. When both assumptions were met, the data were analyzed using the ANOVA test, using Tukey’s HSD test as post hoc. When data was not normal, the non-parametric Kruskal–Wallis test followed by Dunn’s test was used as post hoc. For parameters expressed as a percentage, a regression analysis was conducted assuming a β distribution of the data using the “betareg” package [14], and the “emmeans” package was used for post hoc testing [15]. All statistical analyses were performed using R software version 4.2.2 [16].

### 2.2. RNA Extraction, Library Preparation and Bioinformatics Pipeline

Total RNA was extracted from basal sections (1 cm) of the stems of the microshoots using the “FavorPrep Plant Total RNA Purification Mini Kit (for woody Plant)” kit (Favorgen Biotech corp., Pingtung City, Taiwan). For the transcriptomic analysis, samples were collected at the end of the multiplication cycle and prior to any treatment (Control, CTRL), and at three different times (24 h, 48 h and 72 h) after wounding (W) and wounding plus auxin (IBA 2.5 µm, W + IBA) treatments. RNA was treated with DNAase I, and the quality and quantity were evaluated using a Nanodrop 2000c and a Qubit 4 fluorometer (Thermo Fisher Scientific, Waltham, MA, USA; Figure S1).

The sequencing runs were performed in a MinION device (Oxford Nanopore Technologies, ONT, Oxford, UK) with Flow Cells R9.4.1. To build the transcriptome libraries, the PCR-cDNA Barcoding kit (SQK-PCB109, ONT) was used. After reverse transcription and strand switching, the samples were amplified by PCR for 14 cycles. The sequencing runs were developed for 40 h. Guppy (v3.3.1) was used for basecalling and demultiplexing. The quality of the reads was evaluated with Nanoplot [17], and trimming was developed with Porechop version 0.2.4 (https://github.com/rrwick/Porechop, accessed on 15 March 2023).

The *Quercus suber* draft genome version 1.0 [18] was used as the reference for mapping, which was developed with Minimap2 [19] using the ax-splice option. Assembly and quantification of the libraries were performed with Stringtie 2 [20].

### 2.3. Differential Gene Expression and Pathway Enrichment Analysis

The DESeq2 software version 1.30.1 within the R environment [21] was used to perform the differential expression analysis to detect differentially expressed genes (DEGs) in the comparisons made. On one hand, the W libraries (24 h, 48 h and 72 h) were confronted with the CTRL samples, and similarly, the W + IBA (24 h, 48 h and 72 h) libraries were compared with the CTRL samples. Benjamini and Hochberg’s adjustment of the resulting *p* values was used to establish the False Discovery Rate (FDR), set at *p* < 0.05. Transcripts with a log2 fold change > 0 were established as DEGs in each comparison, comprising the DEGs with respect to the CTRL samples for both treatments (W and W + IBA). For the pathway enrichment analysis, the two groups of DEGs were analyzed with the Kobas tool [22], with information available at the Kyoto Encyclopedia of Genes and Genomes repository.

### 2.4. Gene Expression Analysis

Quantitative real-time PCR experiments were developed to characterize the expression of four transcription factors. The list of primers used for this analysis is given in Supplementary Table S1. The primers were designed with the Primer Designing Tool Primer-BLAST software (https://www.ncbi.nlm.nih.gov/tools/primer-blast/ (accessed on 1 March 2024)). Total RNA was extracted as explained above, and 1 µg of total RNA from each sample was reverse-transcribed using the “High Capacity cDNA Reverse Transcription” kit (Applied Biosystems, Foster, CA, USA). 1 µL of cDNA template (8.33 ng of input RNA) and 2x Power SYBR^®^ Green PCR Master Mix (Applied Biosystems) in a final volume of 20 µL were used for each reaction. The PCR thermal profile used was an initial step of 95 °C/10 min, followed by 40 amplification cycles of 95 °C/15 s, 60 °C/1 min, and a final dissociation protocol to obtain the melting profiles (Figure S1). Three biological and three technical replicates were assessed for each sample. Relative expression values were expressed as fold-change using the comparative CT method (ΔΔCT Method) [23]. The genes *CsACT-2* and *CsELF-1* were used as internal references (Supplementary Table S1).

## 3. Results

### 3.1. Auxin Treatment Optimization

**Figure 1:**
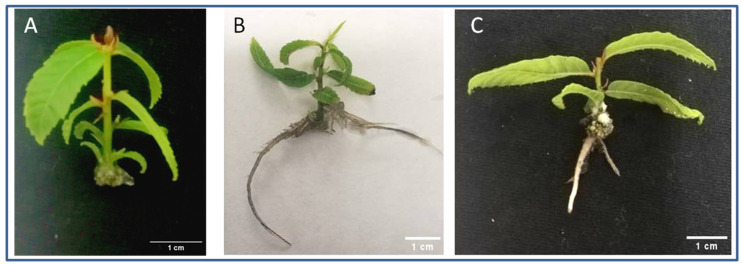
Chestnut P2BS microshoots at the end of the rooting period (30 days). (**A**): Control microshoot showing apical drought and callus at the base of the stem. (**B**): Microshoot treated with IBA 2.5 µm showing good health status and a well-developed root system. (**C**): Microshoot treated with IBA 25 µm showing roots, callus at the base of the stem and apical drought symptoms. Scale bars: 1 cm.

In order to optimize rooting treatments for chestnut microshoots, several exogenous IBA concentrations were tested, ranging from 2.5 to 25 micromoles. All treatments were performed for five days in darkness, and then the microshoots were transferred to the expression media under normal photoperiod conditions and evaluated 30 days after the beginning of the treatments. When IBA was not included in the induction media, microshoots only formed calli in response to wounding (Table 1, Figure 1A).

As shown in Table 1, except for the control, all treatments induced a high rooting rate, almost 100%, with no significant differences among them. Low auxin concentrations induced the formation of adventitious roots at similar rates as those one order of magnitude higher, without significantly modifying the number and length of the roots. However, minor auxin concentrations induced the formation of callus to a significantly lower degree than the 12.5 micromolar concentration, reaching a negligible 2.8% value at 2.5 micromolar (Table 1). A parallel reduction in shoot apical necrosis was also attained for all concentrations tested below 25 micromolar. Interestingly, the rooting kinetics (T50) were not affected by the auxin concentration. Concerning the number of apical or lateral buds of rooted shoots that maintained active growth, higher auxin concentrations were also inversely related to the ability of buds to continue their activity, negatively impacting the ability of the shoot to continue its development. In the case of the 2.5 micromolar treatment, the rate of apical necrosis was very low with normal growth of the shoot apex.

The quality of the shoots was widely impacted by the treatments. While the shoots treated with 25 micromolar concentrations showed callus formation, the highest rate of apical necrosis and an overall inferior health status (Figure 1C), the microshoots treated with a lower amount of auxin presented a healthy aspect (Figure 1B). From these results, the 2.5 micromolar concentration was chosen for further experiments.

### 3.2. Transcriptomic Analysis

**Figure 2:**
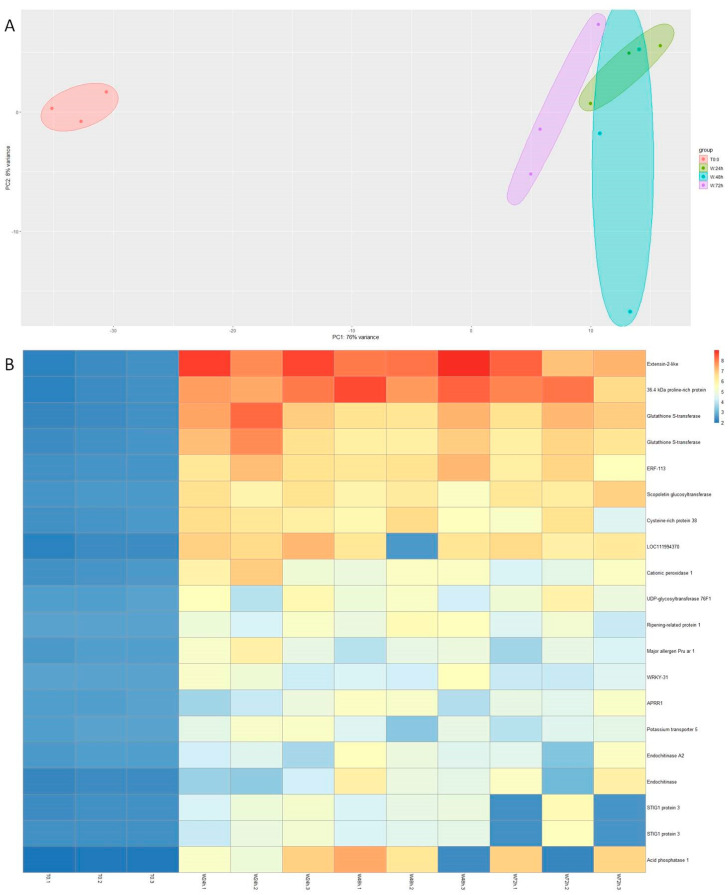
The main results from the wounding transcriptomic analysis. (**A**): Principal component analysis of the sequencing results from the twelve libraries. (**B**): Heatmap showing the 20 top-expressed genes in the wounding samples. Gene IDs are provided in Table S5. T0: control samples at the end of the multiplication cycle. W: Wounded samples.

**Figure 3:**
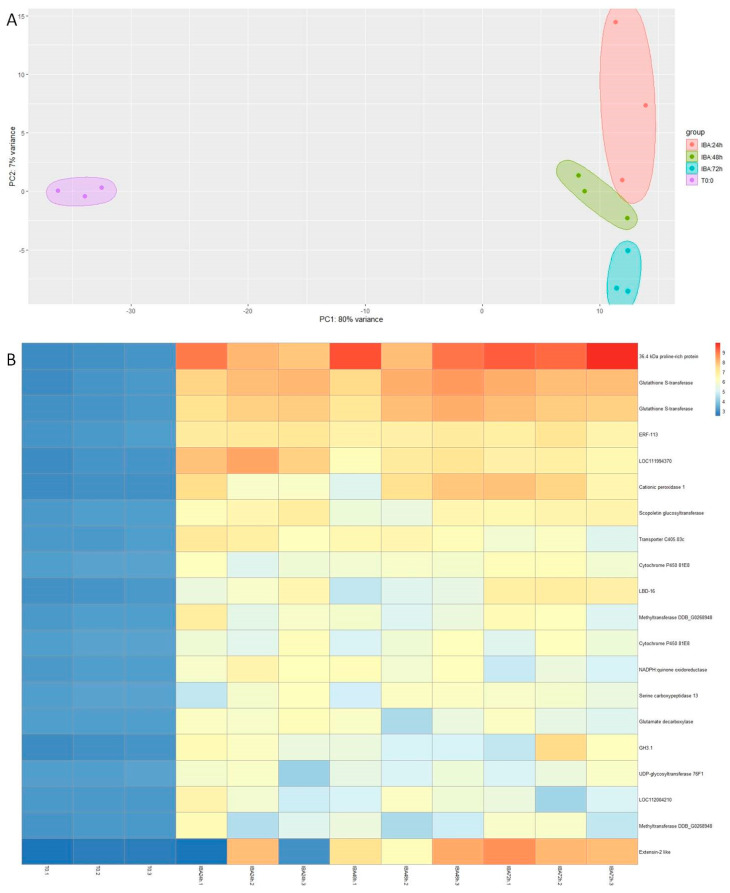
The main results from the wounding + IBA transcriptomic analysis. (**A**): Principal component analysis of the sequencing results from the twelve libraries. (**B**): Heatmap showing the 20 top-expressed genes in the wounding + IBA samples. Gene IDs are provided in Table S6. T0: control samples at the end of the multiplication cycle. IBA: auxin-treated samples.

To characterize the genetic response of chestnut microshoots to the inductive stimuli of wounding (W) and wounding plus auxin (W + IBA), a time series transcriptomics analysis was developed. Samples were collected 24 h, 48 h and 72 h after the beginning of the treatments, and subjected to the sequencing and bioinformatics analysis pipeline. Samples collected at the end of the multiplication period and prior to any treatment were used as controls (CTRL). The sequencing of the 21 samples generated over 27 million reads (16.2 Gb). The mean fragment length was 597 bp and the mean Q score was 9.1 (Supplementary Table S2). The mean percentage of mapping reads to the reference genome was 81.7%. The differential expression analysis performed to detect differentially expressed genes (DEGs) was developed as follows: on the one hand, we confronted the wounding samples (24 h, 48 h and 72 h) with the CTRL samples. Then, W + IBA samples (24 h, 48 h and 72 h) were also compared to the CTRL samples. In this way, genes that were differentially expressed throughout the first 72 h with respect to the starting point could be detected. In the first experiment, the effect of wounding was found to be more relevant than the sample time collection, as seen in the principal component analysis (Figure 2A). A total of 1132 genes were shown to be differentially upregulated in the W samples with respect to the control during the first three days (Supplementary Table S3). The expression of the 20 genes with higher fold change with respect to control samples is shown in Figure 2B, where several transcripts related to oxidative stress, cell wall remodeling and defense were identified (e.g., *Glutathion-S-tranferases*, *Extensin-2-like* and *Endochitinase A2*). Besides, two transcription factors, *CsERF113* and *CsWRKY31*, also showed sustained overexpression with respect to the CTRL samples during the 72 h period (Figure 2B). In the case of the W + IBA treatment, the comparison with the CTRL samples retrieved 1869 DEGs with greater expression in the treated plants (Supplementary Table S4). As in the other case, PCA showed that the treatment had a greater effect on gene expression than the time passed from the beginning of the experiment (Figure 3A). A heatmap showing the top 20 overexpressed genes is shown in Figure 3B. Nine genes were common between this group and the group from the W samples, including *CsERF113*. Other genes overexpressed in the W + IBA top 20 group included auxin-responsive genes (*CsGH3.1*), a transcription factor from the LBD family (*CsLBD16*) and two methyltransferases, among others (Figure 3B).

**Figure 4:**
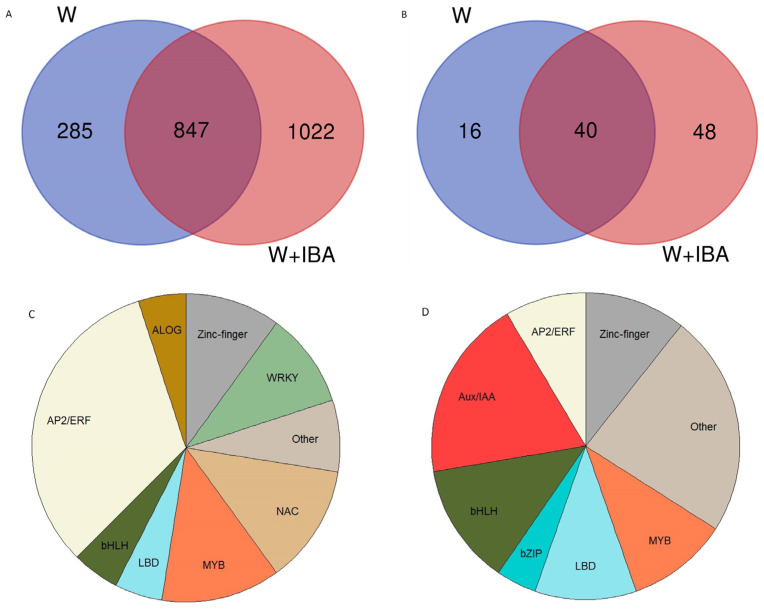
Comparison of the identity of the differentially expressed genes (DEGs) in the wounding (W) and W + IBA libraries. (**A**): Venn diagram showing the common DEGs in the two treatments. (**B**): Venn diagram showing the common transcription factors among the DEGs in the two treatments. (**C**): Family distribution of the transcription factors common to the W and W + IBA treatments. The ID of the genes is provided in Table S7. (**D**): Family distribution of the transcription factors uniquely detected in the W + IBA treatment. The ID of the genes is provided in Table S8.

The identity of the DEGs detected in both experiments was compared, and it was found that almost 75% of the DEGs from the W samples were also overexpressed in the W + IBA plants (Figure 4). Only 285 DEGs were unique to the wounded plants, while over 50% of the DEGs in the W + IBA samples were not overexpressed in the shoots that were only wounded. We then focused on the transcription factors (TFs) identified in each group of DEGs. Fifty-six TFs were identified in the W samples, of which forty were also present in the W + IBA samples (Figure 4B). Of the remaining 16 TFs, at least 9 had a homolog or closely related TF in the W + IBA exclusive group (Supplementary Table S4). Therefore, only 7 TFs were found to be unique to the W samples, including *C. sativa Squamosa Promoter Binding-like 6* and *CsGTE-6 like*. Family classification of the TFs common to the W and W + IBA samples showed that the most abundant group were the TFs belonging to the ethylene responsive AP2/ERF superfamily, with other represented families including MYB and NAC genes (Figure 4C). On the other hand, the most represented families in the 48 TFs exclusively detected in the W + IBA samples were Aux/IAA, LOB domain (LBD) and bHLH, while the AP2/ERF group had a smaller representation (Figure 4D).

**Figure 5:**
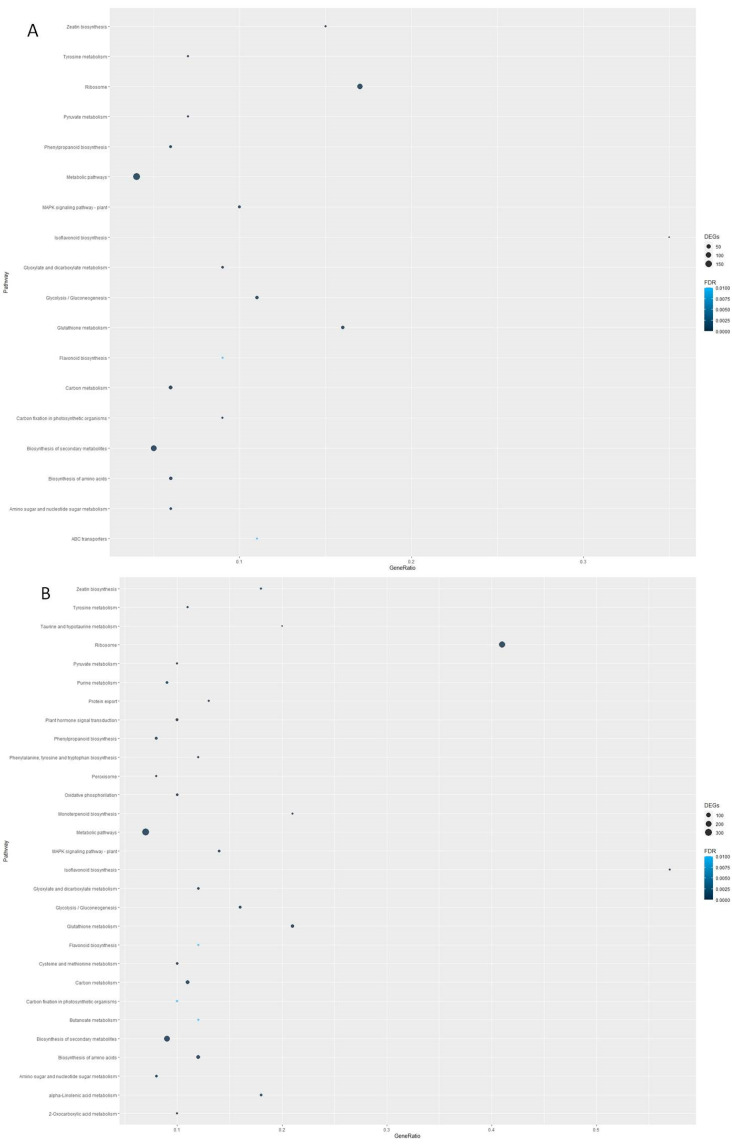
Pathway enrichment analysis of the DEGs detected in the transcriptomic analysis. (**A**): Pathways enriched in the DEGs from the W samples. (**B**): Pathways enriched in the DEGs from the W + IBA samples. FDR: false discovery rate. GeneRatio: number of genes in the DEGs with respect to the total number of genes in the pathway.

To characterize the group of DEGs detected in both comparisons, a pathway enrichment analysis was performed. The DEGs from the W samples were found to be enriched in 18 different pathways, including ribosome, glutathione metabolism, glycolisis/gluconeogenesis and zeatin biosynthesis (Figure 5A). Interestingly, all the pathways but one (ABC transporters) were also detected to be enriched in the DEGs from the W + IBA samples, with similar degrees of enrichment (gene ratio and number of DEGs; Figure 5B). These results suggest that most responses to W are included within the context of the W + IBA treatment. Nonetheless, the different number of DEGs detected in both treatments (1132 vs. 1869) might be influencing the outcome of the analysis. In the W + IBA samples, specific pathways were found to be enriched, including plant hormone signal transduction, peroxisome, monoterpenoid biosynthesis and α-linolenic acid metabolism, which is related to jasmonate synthesis (Figure 5B). Therefore, almost all pathways enriched in the W samples were included within the W + IBA responses, but in the latter, several other pathways related to oxidative stress and specific metabolic processes were also activated.

### 3.3. Expression Analysis of Transcription Factors

**Figure 6:**
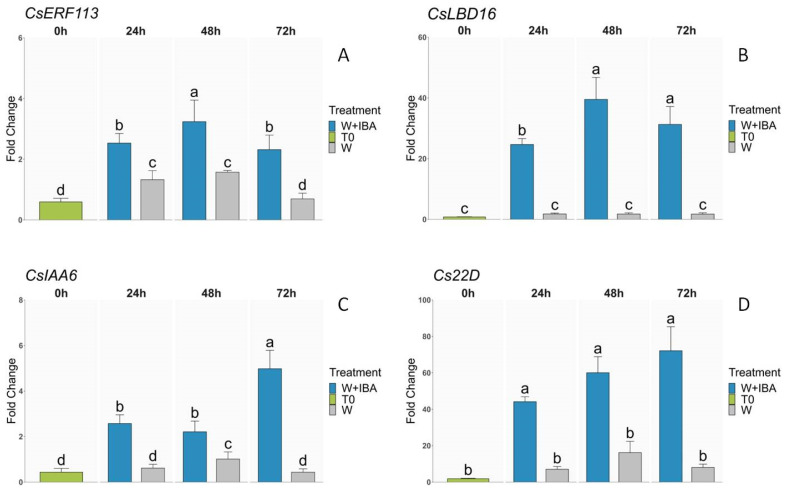
Quantitative real-time expression analysis of transcription factors. (**A**) *CsERF113*. (**B**) *CsLBD16*. (**C**) *CsIAA6*. (**D**) *Cs22D*. Different letters indicate statistical differences at *p* ≤ 0.05. W: Wounded.

To further characterize the responses of chestnut shoots to the two different treatments applied in this study, we selected specific TFs to validate their expression through qPCR. First, we selected two genes that were overexpressed after the two treatments. The ethylene responsive transcription factor *CsERF113-like* was induced significantly in both conditions, but the levels of expression attained were higher during the first 72 h when auxin was present in the medium, particularly at 48 h after the beginning of the treatment (Figure 6A). In the case of *CsLBD16*, a gene from the lateral organ boundaries (LBD) family, the W + IBA treatment induced a high level of expression of this TF from the 24 h time point and up to a 40-degree fold change at 48 h (Figure 6B). In the W samples, according to the qPCR analysis, there was no induction in the expression of the gene despite its detection in the transcriptomics analysis (Table S7). In the case of the TFs exclusively detected in the W + IBA samples, we chose two genes from the Aux/IAA family of auxin-responsive genes, as this family was the most represented in this group of genes (Figure 4D). *CsIAA6* was induced by wounding only at 48 h, while at 24 h and 72 h the level of expression was not significantly different from the CTRL (Figure 6C). However, when IBA was included in the treatments, expression of the gene was clearly induced during the 72 h, regarding the control and W samples, with the highest induction at 72 h. In all time points, the expression in the W + IBA samples was significantly higher than in the W samples (Figure 6C). For the *Cs22D* gene, a similar pattern was detected. In the W samples, levels of expression were greater than in the CTRL; however, the combined analysis of the expression levels of all samples resulted in no significant differences (Figure 6D). The similarity in the pattern can be found in the higher expression in the W samples at 48 h. In any case, the expression levels in the W samples were much lower than those reached in the presence of auxin, with a sustained increase in expression that reached its highest point at 72 h (as with *CsIAA6*; Figure 6C,D). Lower levels of expression in the W samples of *CsIAA6* at 72 h and of *Cs22D* at 24 h might explain why these two TFs were not detected as DEGs in the W samples. Overall, qPCR analysis suggests that these four TFs are induced by the combined effect of wounding and auxin, although the former also influences the expression of some of these genes.

## 4. Discussion

The development of improved protocols for vegetative propagation of forest species is essential for the profitability and sustainability of related industries. Optimized protocols might help increase savings by reducing the amount of chemicals needed to induce AR and by ameliorating the health of the plants at the end of the rooting period. On the other hand, a greater understanding of the molecular basis of this process might help develop better protocols suitable for a greater number of genotypes and ontogenetic states, thus increasing the convenient number of varieties that can be used for commercial purposes. Moreover, it is widely accepted that resistance to several biotic and abiotic menaces has a genetic basis. Therefore, it is necessary to increase the number of suitable genotypes that can be proliferated by these means in order to increase the available genetic pool of material. Successful chestnut vegetative propagation relies on the application of exogenous auxin, mainly IBA, with the concomitant employment of a wounding stress being necessary for the successful responses of cuttings and microshoots, as with cuttings from other species [24]. Two different strategies have usually been applied: a short treatment with a high concentration of auxin or a longer inductive period with a lower amount of auxin, providing contrasting results according to genotypes and the ontogenetic state of the materials (reviewed in [4]). In a previous study, modified auxin treatments were tested on the material used in this study, leading to improved responses in terms of microshoot health without diminishing rooting rates [9]. These findings led to the design of experiments with even lower amounts of auxin that have proven successful (Table 1). However, the presence of exogenous auxin, even at low concentrations, is required for the induction and development of Ars, as control shoots did not root. The induction of ARs relies on the creation of auxin gradients in the tissues, with the differences in auxin content between neighboring tissues leading to the induction of specific auxin-related gene expression [24,25]. For this purpose, the activity of auxin polar transporters, particularly those of the PIN family, seems crucial. In a previous study, the ability of mature chestnut shoots to improve their rooting responses was linked to a greater expression of *CsPIN1* [26]. Within the group of DEGs detected after the W + IBA treatment, several auxin transporters were identified (*CsPIN3*, *CsPIN7* and *CsAEC1*), but only *CsPIN3* was identified in the W samples (Supplementary Table S3). Local auxin biosynthesis and transport modulation are common responses at the wound site, as basipetally transported auxin accumulates at the wound site, while local auxin biosynthesis is also induced to trigger cellular reprogramming [2,7,27]. However, low amounts of exogenous auxin seem to induce the expression of the set of genes needed to establish the correct auxin gradients for the development of ARs. Moreover, it was also found that lowering the exogenous auxin inputs improved the overall health status of the shoots at the end of the rooting cycle. Apical drought is believed to be a consequence of an imbalance in the homeostasis of cytokinins [28], and their crosstalk with auxins has also been proposed as a plausible explanation [4]. Apparently, less auxin in the medium improves this balance and helps in the correct deployment of apical bud activity. Besides, it was found that with decreasing auxin concentrations, apical or lateral buds were keen to keep active growth, the latter when the apical bud was unable to, leading to continuous growth of the shoots.

In response to wounding stress, chestnut shoots generate a callus tissue, an asymmetric disorganized structure whose induction also requires cellular reprogramming of the neighboring cells, the initiation of cell division and is controlled by auxin signaling [7,29], sharing genetic elements with the root developmental pathway [30]. This process of regeneration is essential to preserving plant integrity, with the crosstalk between hormone and stress signaling establishing the fate of the cells at the wound site [7]. Developmental pathways that drive the generation of this tissue have been explored in recent years, proving that it is a common response in plants related to several regeneration mechanisms and that the genetic program depends on the nature of the original cells and the given conditions [31]. Our results support the idea of shared mechanisms with the root developmental pathway, according to the number of common DEGs found between the two applied treatments and the similarity in the enriched pathways in the two analyzed conditions. Indeed, a significant number of TFs were induced by W and W + IBA, suggesting that a relevant number of the molecular processes needed for the generation of ARs are already induced by wounding, and indicating that the AR developmental pathway could be interpreted as an expanded wounding response. The wounding-related reprogramming fails to mark cells as rooting initials, which is only achieved in the presence of exogenous auxin.

Analysis of the expression of selected TFs showed that specific levels of the transcripts are needed for the successful development of roots. *CsERF113-like* and *CsLOB16* were shown to be induced by both treatments through transcriptomics analysis. However, the qPCR results showed that higher levels of expression were achieved in the presence of auxin. *CsERF113* belongs to the family X of ERFs (ERF109-ERF115), that have been linked to regeneration processes [32]. In poplar, *PdeERF114* was shown to govern the formation of callus together with *PdeWRKY75*, both influencing the expression of genes linked to H_2_O_2_ accumulation and cell wall remodeling, and specifically linking the activity of the gene with callus formation [33]. Another member of this subgroup, *AtERF115*, is expressed in wound site adjacent cells, conferring them stem-cell identity that, together with auxin active signaling, triggers regenerative divisions (reviewed in [8]). Indeed, in combination with *AtARF5*, *AtERF115* is involved in the regeneration of the root meristem after root-tip excision [7]. Therefore, in chestnut, low inputs of exogenous auxin are able to modify *CsERF113-like* expression levels to overcome the callus-induced program resulting in the formation of ARs. Similarly, the activity of *LBD* genes has also been linked with the formation of calli and ARs. Several genes from the LBD family (*LBD16*, *LBD17*, *LBD18* and *LBD29*) have been shown to induce the formation of calli in Arabidopsis [34]. However, *LBD16* is linked with the acquisition of pluripotency and the generation of both calli and ARs [7]. Indeed, authors have suggested that the combined expression of *LBD16* and *LBD29* is enough to tag specific cells as root founder cells in de novo root regeneration [35], and this specific signaling pathway in response to auxin has also been found in apple [36]. *LBD29* genes have been shown to govern gene expression related to the modification of the cell walls [37]. The transcriptomic analysis revealed three *CsLBD29* homologs induced in response to auxin (Table S8). Together with the high level of expression attained by *CsLBD16*, the present results suggest that the aforementioned module might also be active in chestnut. However, qPCR analysis did not show a significant induction in the expression of *CsLBD16* in response to wounding. Therefore, *CsERF113* is responsive to both treatments, while the response of *CsLBD16* to wounding will require further analysis. Nonetheless, in the presence of exogenous auxin, their expression seems to allow for specific interactions driving the formation of ARs.

In the set of TFs exclusively detected in W + IBA-treated plants, the IAA family was the most abundant. This family of auxin-responsive genes codes for short-lived proteins that work as transcriptional repressors, and it is at the core of the canonical auxin signaling pathway. Their domain structure allows them to homodimerize or, more relevantly, heterodimerize with members of the Auxin Response Factor (ARF) family, thus impeding their activity as modulators of auxin-responsive genes [38], also influencing the AR process, at least in Arabidopsis (reviewed in [5]). In Eucalyptus, *EgrIAA13* was shown to interact with four different ARFs, directly impacting the xylogenesis process [39]. Nonetheless, Aux/IAA proteins have been shown to interact with many other TFs and proteins, and they have been proposed to work as sensors of auxin levels, influencing several responses in plants [40]. *Cs22D* showed an extraordinary increase and sustained induction that may counteract its expected short half-life. This gene, a close homolog of *AtIAA4*, might be involved in the modulation of the auxin response to induce ARs. In *Camellia sinensis*, *CsiIAA4* directly interacts with *CsiMYB4a* to regulate stamen growth, and the interaction prevents the degradation of *CsiIAA4* [41]. It is possible that unknown interactions of *Cs22D* with other TFs prevent its degradation and define its specific roles in AR. On the other hand, *CsIAA6* was transiently induced by wounding, suggesting that this treatment provokes the local accumulation of auxin. Induction of *CsIAA6* in response to IBA was more modest than in the case of *Cs22D*, attaining its higher level of expression at 72 h. In Arabidopsis, *AtIAA6* has been proposed to interact with different members of the auxin signaling pathway to control AR formation [42]. Overall, *Aux/IAA* genes seem to present a plethora of interactions to translate auxin levels to specific developmental responses according to tissue and time context [40]. Further exploration of these interactions will clarify the role played by *Cs22D* and *CsIAA6* in AR.

## 5. Conclusions

An optimized protocol for the induction of ARs was developed for chestnut microshoots. The protocol implies the application of small amounts of auxin to obtain rooted plantlets with an overall good health status. A transcriptomic analysis of the responses of the microshoots to this low-auxin treatment and to an auxin-free treatment provided significant results concerning the molecular responses of chestnut during the AR process. A relevant number of molecular mechanisms and genes were found to be common for both treatments. However, specific expression of selected transcription factors was only attained in the presence of exogenous auxin, thus suggesting that the wounding response is integrated within the exogenous-auxin-triggered response to induce roots.

## Figures and Tables

**Table 1:** Results of the different auxin treatments on the rooting performance of P2BS microshoots.

Clone	IBA (μm)	Rooting (%)	Root Number	Root Length (cm)	Callus (%)	Apical Necrosis (%)	T50	Active Growth (%)
P2BS	0	0 b	-	-	100 ± 0 a	83.3 ± 4.1 a	-	-
	2.5	97.2 ± 4.8 a	3.0 ± 1.4 a	4.4 ± 1.8 a	2.8 ± 1.4 c	2.8 ± 4.8 e	8 a	100 ± 0 a
	5	97.2 ± 4.8 a	3.7 ± 1.0 a	4.1 ± 0.8 a	2.8 ± 1.4 c	27.7 ± 4.1 d	7 a	69.9 ± 5.8 b
	10	94.4 ± 4.8 a	4.3 ± 1.9 a	4.2 ± 1.5 a	5.3 ± 2.1 c	27.7 ± 4.1 d	8 a	30.3 ± 4.6 c
	12.5	94.4 ± 9.3 a	4.2 ± 1.9 a	4.1 ± 1.2 a	27.7 ± 4.6 b	55.3 ± 9.2 c	8 a	20.4 ± 11.7 d
	25	93.2 ± 4.6 a	4.0 ± 1.8 a	3.5 ± 1.4 a	100 ± 0 a	72.0 ± 5.1 b	7 a	14.0 ± 5.2 d

IBA: Indole-butyric acid. T50: days passed until the rooting of half of the microshoots. Different letters indicate significant statistical differences (ANOVA, *p* < 0.05).

## Data Availability

The FastQ files generated for each of the samples are available at the NCBI repository (https://www.ncbi.nlm.nih.gov/bioproject, accessed on 13 March 2024) under accession ID PRJNA1083332.

## Supplementary Materials

The following supporting information can be downloaded at: https://www.mdpi.com/article/10.3390/genes15030388/s1. Table S1: list of primers used in the qRT-PCR experiments; Table S2: overall parameters of the transcriptomic datasets; Table S3: list of DEGs in the Wounding samples; Table S4: list of DEGs in the Wounding + IBA samples; Table S5: gene IDs for DEGs in Figure 2B; Table S6: gene IDs for DEGs in Figure 3B; Table S7: list of transcription factors common to the Wounding and Wounding + IBA groups of DEGs; Table S8: list of transcription factors uniquely detected in the Wounding + IBA group of DEGs; Figure S1: quality assessment of RNA samples for transcriptomic analysis and melting curves of qPCR products.
