# Supplementary material for: Wounding-Related Signaling Is Integrated within the Auxin-Response Framework to Induce Adventitious Rooting in Chestnut
Source: Genes (Basel). 2024 Mar 21;15(3):388. doi: 10.3390/genes15030388 (PMC10970416; doi:10.3390/genes15030388)
Supplement: Supplementary file 1 [file genes-15-00388-s001.zip › Supplemental Figure S1.pptx]

## Slide 1
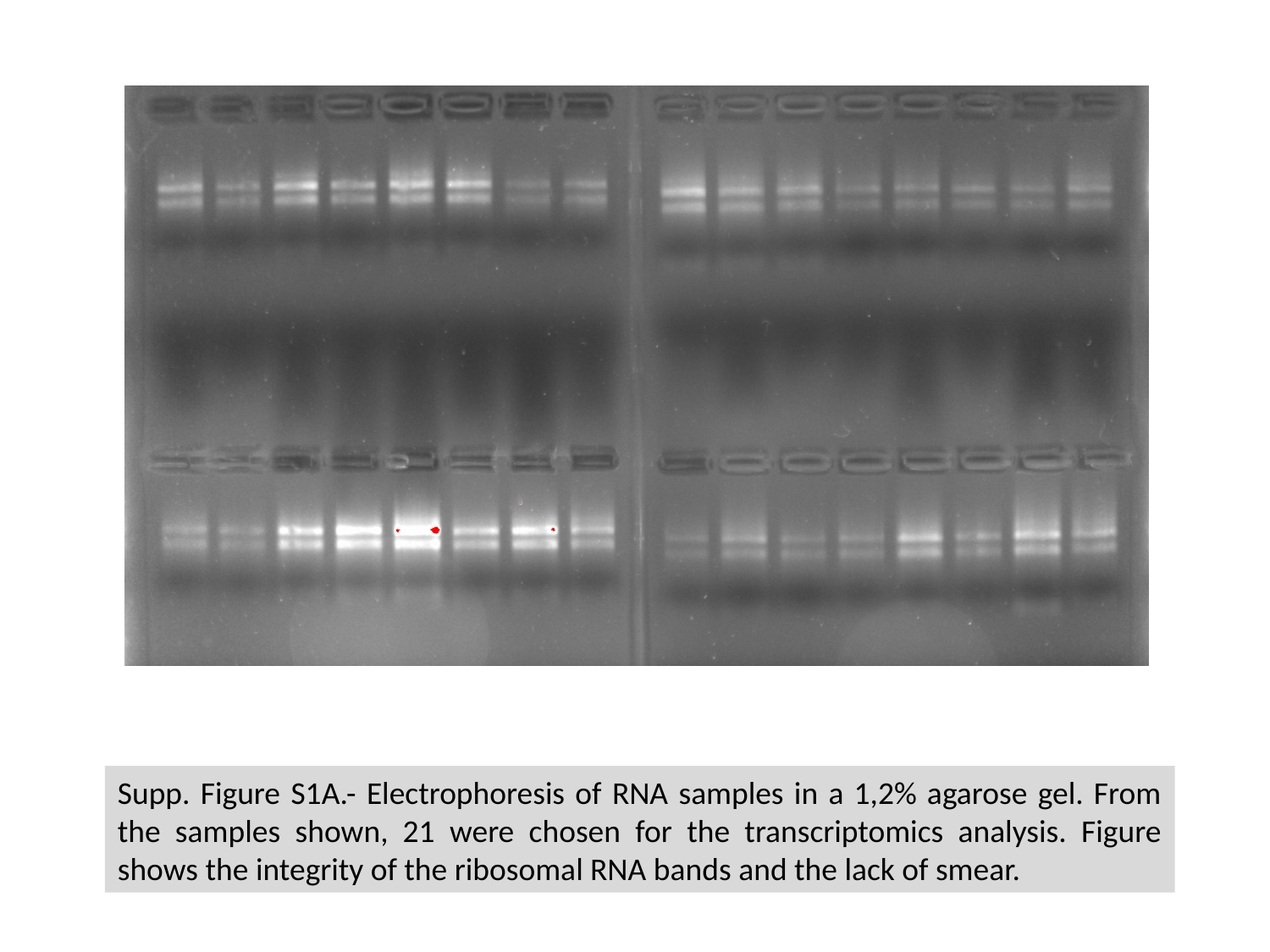

Supp. Figure S1A.- Electrophoresis of RNA samples in a 1,2% agarose gel. From the samples shown, 21 were chosen for the transcriptomics analysis. Figure shows the integrity of the ribosomal RNA bands and the lack of smear.

## Slide 2
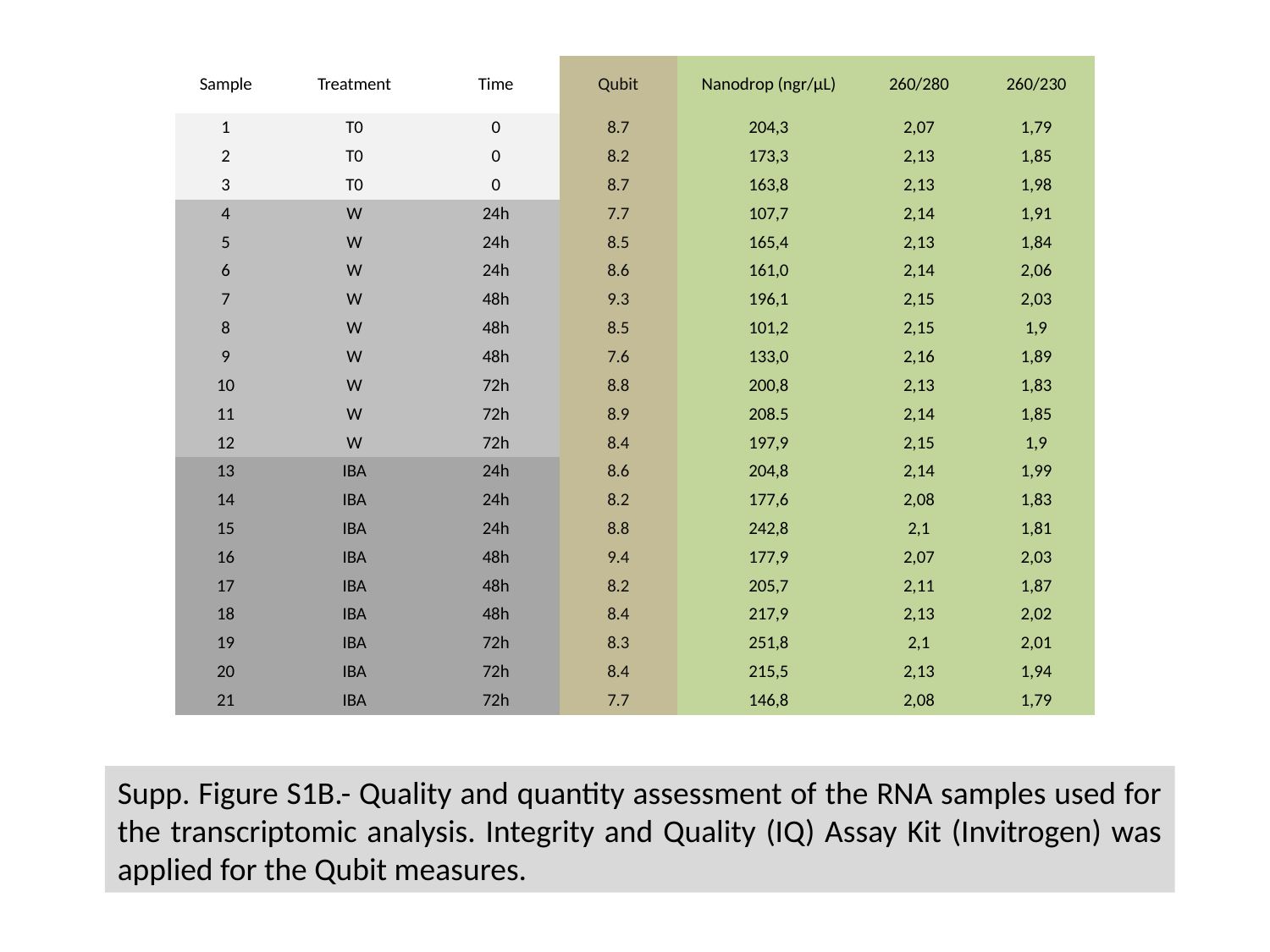

| Sample | Treatment | Time | Qubit | Nanodrop (ngr/µL) | 260/280 | 260/230 |
| --- | --- | --- | --- | --- | --- | --- |
| 1 | T0 | 0 | 8.7 | 204,3 | 2,07 | 1,79 |
| 2 | T0 | 0 | 8.2 | 173,3 | 2,13 | 1,85 |
| 3 | T0 | 0 | 8.7 | 163,8 | 2,13 | 1,98 |
| 4 | W | 24h | 7.7 | 107,7 | 2,14 | 1,91 |
| 5 | W | 24h | 8.5 | 165,4 | 2,13 | 1,84 |
| 6 | W | 24h | 8.6 | 161,0 | 2,14 | 2,06 |
| 7 | W | 48h | 9.3 | 196,1 | 2,15 | 2,03 |
| 8 | W | 48h | 8.5 | 101,2 | 2,15 | 1,9 |
| 9 | W | 48h | 7.6 | 133,0 | 2,16 | 1,89 |
| 10 | W | 72h | 8.8 | 200,8 | 2,13 | 1,83 |
| 11 | W | 72h | 8.9 | 208.5 | 2,14 | 1,85 |
| 12 | W | 72h | 8.4 | 197,9 | 2,15 | 1,9 |
| 13 | IBA | 24h | 8.6 | 204,8 | 2,14 | 1,99 |
| 14 | IBA | 24h | 8.2 | 177,6 | 2,08 | 1,83 |
| 15 | IBA | 24h | 8.8 | 242,8 | 2,1 | 1,81 |
| 16 | IBA | 48h | 9.4 | 177,9 | 2,07 | 2,03 |
| 17 | IBA | 48h | 8.2 | 205,7 | 2,11 | 1,87 |
| 18 | IBA | 48h | 8.4 | 217,9 | 2,13 | 2,02 |
| 19 | IBA | 72h | 8.3 | 251,8 | 2,1 | 2,01 |
| 20 | IBA | 72h | 8.4 | 215,5 | 2,13 | 1,94 |
| 21 | IBA | 72h | 7.7 | 146,8 | 2,08 | 1,79 |
Supp. Figure S1B.- Quality and quantity assessment of the RNA samples used for the transcriptomic analysis. Integrity and Quality (IQ) Assay Kit (Invitrogen) was applied for the Qubit measures.

## Slide 3
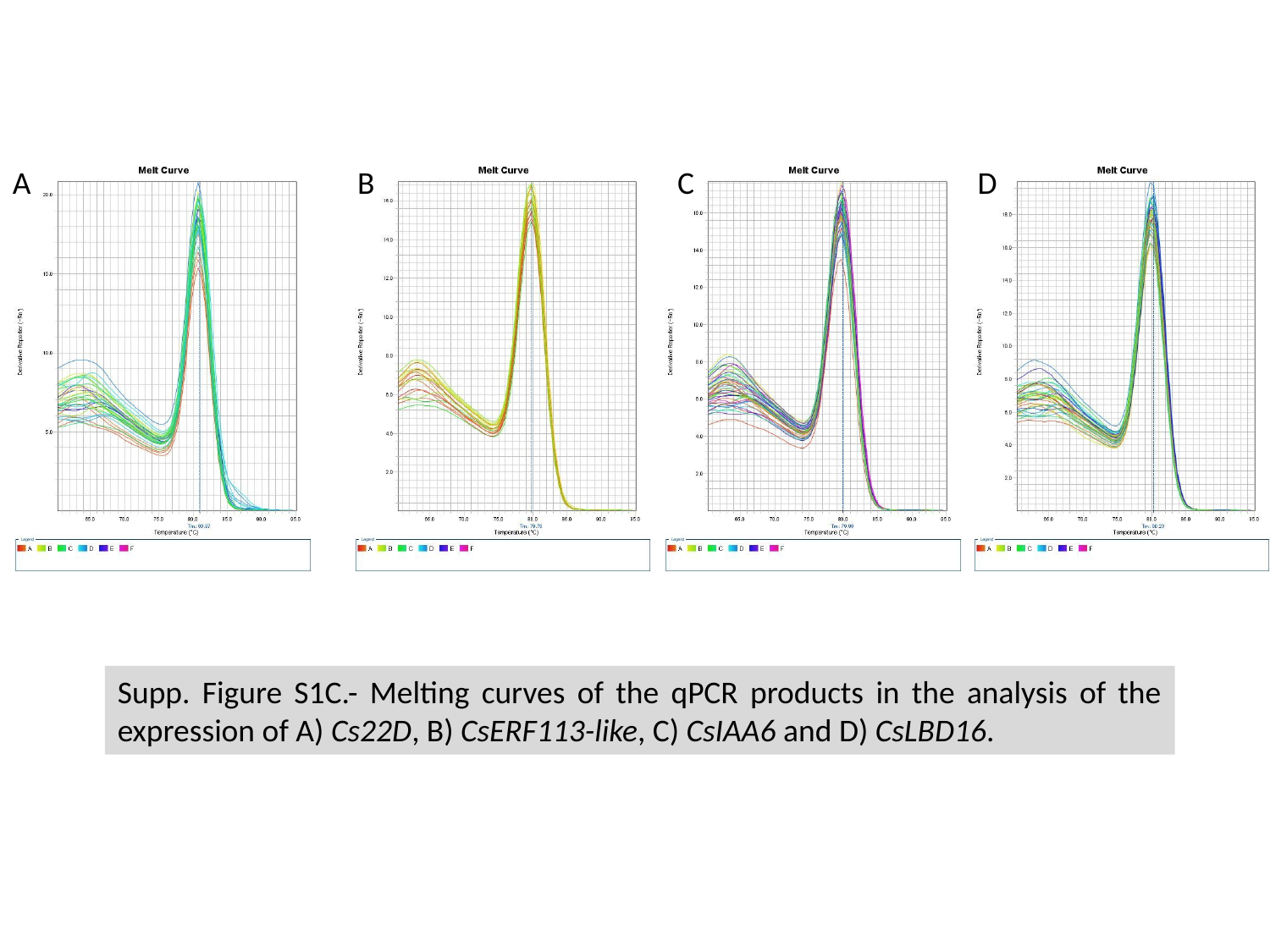

A
B
C
D
Supp. Figure S1C.- Melting curves of the qPCR products in the analysis of the expression of A) Cs22D, B) CsERF113-like, C) CsIAA6 and D) CsLBD16.
